# Ectopic RNF168 expression promotes break-induced replication-like DNA synthesis at stalled replication forks

**DOI:** 10.1093/nar/gkaa154

**Published:** 2020-03-17

**Authors:** John J Krais, Neil Johnson

**Affiliations:** Molecular Therapeutics Program, Fox Chase Cancer Center, Philadelphia, PA 19111, USA

## Abstract

The RNF168 E3 ubiquitin ligase is activated in response to double stranded DNA breaks (DSBs) where it mono-ubiquitinates γH2AX (ub-H2AX). RNF168 protein expression and ubiquitin signaling are finely regulated during the sensing, repair and resolution of DNA damage in order to avoid excessive spreading of ubiquitinated chromatin. Supra-physiological RNF168 protein expression levels have been shown to block DNA end resection at DSBs and increase PARP inhibitor (PARPi) sensitivity. In this study, we examined the impact of ectopic RNF168 overexpression on hydroxyurea (HU)-induced stalled replication forks in the setting of BRCA1 deficiency. Surprisingly, RNF168 overexpression resulted in the extension of DNA fibers, despite the presence of HU, in BRCA1 deficient cells. Mechanistically, RNF168 overexpression recruited RAD18 to ub-H2AX at HU-induced DNA breaks. Subsequently, a RAD18-SLF1 axis was responsible for initiating DNA synthesis in a manner that also required the break-induced replication (BIR) factors RAD52 and POLD3. Strikingly, the presence of wild-type BRCA1 blocked RNF168-induced DNA synthesis. Notably, BIR-like repair has previously been linked with tandem duplication events found in *BRCA1*-mutated genomes. Thus, in the absence of BRCA1, excessive RNF168 expression may drive BIR, and contribute to the mutational signatures observed in *BRCA1*-mutated cancers.

## INTRODUCTION

Homologous recombination (HR) DNA repair is paramount for maintaining the genome integrity of normal cells and tissues (1). Cancer cells demonstrate high levels of genomic instability, often arising as a consequence of poorly coordinated DNA replication, which gives rise to spontaneous breaks that are repaired by low fidelity pathways (2,3). Because BRCA1 plays a critical role in DNA end resection and RAD51 loading, *BRCA1*-mutated cancers are unable to effectively carry out HR repair and rely on alternative pathways, such as non-homologous end joining (NHEJ), single-strand annealing (SSA) and microhomology-mediated end joining (MMEJ) (1,4,5). These pathways are able to process and repair two-ended DSBs. However, the degree to which each of these are involved in the repair of one-ended DSBs that arise during DNA synthesis is unclear.

One-ended DSBs arise at stalled replication forks and are typically repaired by HR, but in the absence of BRCA1, BRCA2 or RAD51, long-tract gene conversion (LTGC) repair events become dominant (6,7). LTGC is error-prone and may cause tandem duplications (TDs) (8). The TD Group 1 mutational signature, which is characterized by abundant small (∼10 kb) TDs with microhomology breakpoints, is observed in *BRCA1* mutant cancers (8–10). LTGC may be analogous to BIR in yeast. BIR has been shown to occur in mammalian cells that have oncogene-induced replication stress and is dependent on POLD3 and RAD52 activity (11,12). The molecular processes involved in break detection and signaling that control the initiation of BIR in mammalian cells are not well defined.

RNF168 localizes to DSBs where it ubiquitinates histone γH2AX at K13/15 (13). The latter serves as a recruitment scaffold for ubiquitin-binding proteins including 53BP1, RAD18 as well as RNF168 itself (14,15). 53BP1 is an inhibitor of DNA end resection and HR (16,17), while RAD18 is an E3 ubiquitin ligase that mono-ubiquitinates PCNA and activates translesion synthesis (TLS) (18). Independent from PCNA ubiquitination, RAD18 has also been shown to promote DNA synthesis and recombination in a manner that is dependent on its ability to localize to ubiquitin sites and complex with SLF1 (19).

Subsets of *BRCA1* mutant cancers reported in the TCGA database show increased *RNF168* mRNA expression (20). Significantly, supra-physiologic RNF168 levels were previously shown to influence DSB repair pathway dynamics, with ectopic RNF168 overexpression reducing DNA end resection and increasing PARPi cytotoxicity in BRCA1 deficient cells (20,21). In this study, we asked how RNF168 overexpression impacts DNA replication fork dynamics in the setting of BRCA1 deficiency.

## MATERIALS AND METHODS

### cDNA constructs and lentivirus production

RNF168 and ub-H2AX cDNA constructs were generously provided by Dr Daniel Durocher and Dr Thanos Halazonetis, respectively. POLD3 cDNA was obtained from GeneCopoeia (catalog# GC-Y2063-CF) and RAD18 cDNA from Addgene (catalog# 68827). The cDNAs were PCR amplified and ligated into the Gateway entry vector pENTR1A (ThermoFisher Scientific) and shuttled into pCW57.1 or PLX304 using the LR Clonase II Enzyme Mix (ThermoFisher Scientific). Lentivirus was produced and cells were selected with 4 μg/ml puromycin for pCW57.1 or 4 μg/ml blasticidin for PLX304. Expression in pCW57.1 is doxycycline inducible, which was added to cultures at 4 μg/ml 72 h prior to experiments. *BRCA1* cDNA was cloned into pDest-IRES-GFP, cells transduced with lentivirus and selected for GFP expression by FACS. To generate lentivirus HEK293T cells were transfected with pxPAX2 packaging plasmid, VSV-G envelope plasmid and cDNA containing expression plasmids using TransIT-LT1 transfection reagent. Cell culture media was changed 18 h post-transfection to DMEM + 30% FBS and was collected after 48 h then pushed through a 0.45 μm filter. Cell lines were infected with lentivirus in polybrene containing media and were maintained in media containing TET-free FBS (Atlanta Biologicals).

### Cell culture

Cell lines were obtained from ATCC or Asterand and cultured as previously described (22,23). All cell lines containing doxycycline inducible constructs were maintained in media containing 10% TET-free FBS and expression induced with 4 μg/ml doxycycline 72 h prior to experiments.

### Western blotting

Nuclear extracts were obtained using the NE-PER Nuclear and Cytoplasmic Extraction Kit (Thermo Scientific) and whole cell extracts were generated using RIPA buffer with protease and phosphatase inhibitors added. Proteins were separated by sodium dodecyl sulphate (SDS)-polyacrylamide gelelectrophoresis and transferred to a polyvinylidene fluoride (PVDF) membrane. Membranes were blocked with 5% nonfat milk in phosphate-buffered saline tween 20 (PBST) at room temperature for 1 h. Primary antibodies were incubated overnight at 4° and horseradish peroxidase (HRP)-conjugated secondary antibodies were incubated for 1 h at room temperature. The following primary antibodies were used: BRCA1 (EMD Millipore, catalog# OP92), RNF168 (EMD Millipore, #06-1130-I), Tubulin (Cell Signaling, catalog# 2148), GFP (Santa Cruz Biotechnology, catalog# sc-9996), RFP (ChromoTek, catalog# 6g6-20), FLAG (Cell Signaling, catalog# 14793 and Sigma Aldrich, catalog# F1804), phospho-Chk1 (Cell Signaling, catalog# 2344), Chk1 (Cell Signaling, catalog# 2360), POLD3 (Bethyl Laboratories, A301-244A), PALB2 (Bethyl Laboratories, catalog# A301-246A), BRCA2 (Bethyl Laboratories, catalog# A303-435A), RAD51 (Santa Cruz Biotechnology, catalog# sc-8349), RAD18 (Bethyl Laboratories, catalog# A301-340A), 53BP1 (Cell Signaling, catalog# 4908).

### DNA fiber assays

Exponentially growing cells were incubated with 50 μM CldU, 250 μM IdU and 4 mM hydroxyurea for intervals specified for each experiment and washed 3× with phosphate-buffered saline (PBS) between each incubation step. For siRNA experiments, cells were reverse transfected 72 h prior to incubation with CldU, IdU and HU. Cells were collected, resuspended in PBS, then lysed on slides (Superfrost Plus microscope slides, Fisher Scientific) with 200 mM Tris–HCl, 50 mM ethylenediaminetetraacetic acid, 0.5% SDS, pH 7.4 buffer. Slides were tilted 60° to spread fibers, air dried, fixed in 3:1 methanol: acetic acid for 10 min, and stored overnight at −20°. DNA fibers were denatured in 2.5 M HCl for 2.5 h, washed with PBS, then blocked with 2% bovine serum albumin (BSA) in PBST for 40 min at room temperature. Slides were incubated with primary antibodies recognizing CldU (Abcam, catalog# ab6326, dilution 1:300) and IdU (BD Biosciences, catalog# 347580, dilution 1:100) for 2.5 h at room temperature, washed, then incubated with AlexaFluor488 and AlexaFluor594 conjugated secondary antibodies (ThermoFisher Scientific, catalog# A11062 and A21470, dilutions 1:300) for 1 h at room temperature. Images were acquired with a Nikon NIU Upright Fluorescence microscope and fiber lengths measured using ImageJ software.

### siRNA experiments

siRNA experiments were conducted by reverse transfection with Lipofectamine RNAimax (ThermoFisher Scientific, catalog# 13778030) according to manufacturer instructions. The following siRNA were used: AllStars Negative Control siRNA (scrambled control) (Qiagen, catalog# SI03650318), 53BP1 #1 GAAGGACGGAGUACUAAUAUU, 53BP1 #2 GCUAUAUCCUUGAAGAUUUUU, 53BP1 #3 GAGCUGGGAAGUAUAAAUUUU, 53BP1 #4 GGACUCCAGUGUUGUCAUUUU, 53BP1 SmartPool (Dharmacon), RNF168 #1 GACACUUUCUCCACAGAUAUU, RNF168 #2 CAGUCAGUUAAUAGAAGAAAUU, BRCA1 #1 ACCAUACAGCUUCAUAAAUAAUU, BRCA1 #2 CAGCAGUUUAUUACUCACUAAUU, MUS81 #1 UCUACCGGGAGCACCUGAAUCCUAA, MUS81 #2 GCAGGAGCCAUCAAGAAUAUU, SLX4 #1 AAACGUGAAUGAAGCAGAAUU, SLX4 #2 UUUGGAUGAAGAUUUCUGAGAUCUGUU, RAD52 #1 GGUCAUCGGGUAAUUAAUCUU, RAD52 #2 GGCCCAGAAUACAUAAGUAUU, POLD3 #1 ACGAAAACGCGUACUAAAAUU, POLD3 #2 GGCAUUAUGUCUAGGACUAUU, POLQ #1 CAACAACCCUUAUCGUAAAUU, POLQ #2 CGACUAAGAUAGAUCAUUUUU, BRCA2 #1 GCCCGAUUCCGUAUUGGUAUU, BRCA2 #2 GCUUAACCUUUCCAGUUUAUU, RAD51 #1 CCAACGAUGUGAAGAAAUUUU, RAD51 #2 UCUUCCUGUUGUGACUGCCAGGAUAUU, PALB2 #1 GGUGUACAUAAAGCUUCAAUU, PALB2 #2 GGAUAUAUUGGGCCUCUUAUU, RAD18 #1 ACUCAGUGUCCAACUUGCU, RAD18 #2 GCUCUCUGAUCGUGAUUUA, RAD18 #3 GCAAGAAACAGUUGAGUUAUU, RAD18 #4 GGUUGUUGCCCGAGGUUAAUU, SLF1 SmartPool (Dharmacon), RAD6A GAACAAGCUGGCGUGAUU, RAD6B CAAACGAGAAUAUGAGAAA. RAD6A and RAD6B siRNA were pooled together for RAD6 knockdown.

### Immunofluorescence

Cells were grown in 10 μM EdU for the indicated periods of time then subject to pre-extraction in cold cytoskeleton buffer (10 mM Pipes pH 6.8, 100 mM NaCl, 300 mM sucrose, 3 mM MgCl2, 1 mM EGTA, 0.5% Triton X-100) for 5 min on ice followed by 5 min incubation with cold cytoskeleton stripping buffer (10 mM Tris HCl pH 7.4, 10 mM NaCl, 3mM MgCl2, 1% Tween 40(v/v), 0.5% sodium deoxycholate). Cells were then fixed with 4% paraformaldehyde and permeabilized with 1% Triton-X100 in PBS for 10 min. For EdU foci, following permeabilization the cells were washed with 2% BSA in PBS, and a click chemistry reaction (2 mM copper sulfate, 1 mM azide-fluor 488, 10 mM sodium ascorbate) performed for 30 min. Cells were then washed again with 2% BSA in PBS followed by 3× PBS washes and coverslips mounted with mounting media containing DAPI (Vector Laboratories Inc.). For RAD18 foci, following permeabilization cells were incubated overnight at 4° with primary antibody (Bethyl Laboratories, catalog# A301-246A) in 5% goat serum, washed and incubated with secondary antibody for 1 h at room temperature prior to mounting coverslips. Z-stack images were captured using a Nikon NIU Upright Fluorescence microscope and generated images using Nikon NIS Elements software. Foci quantifications were reported as percent foci positive nuclei or foci count per nuclei. Percent positivity was determined based on nuclei containing five or more foci and assessed for five fields of view for a minimum of 200 cells per condition.

### Statistical analysis

Statistical tests were performed as indicated in each figure legend. DNA fiber ratio analyses compared the median values of non-parametric samples as indicated using the Mann–Whitney test with Graphpad Prism software. Statistical significance of foci differences were assessed by two-tailed unpaired t test using the comparisons indicated in each figure. Statistically significant *P*-values are shown in figure legends.

## RESULTS

### Ectopic RNF168 produces unlabeled gaps during HU treatment

**Figure 1.**
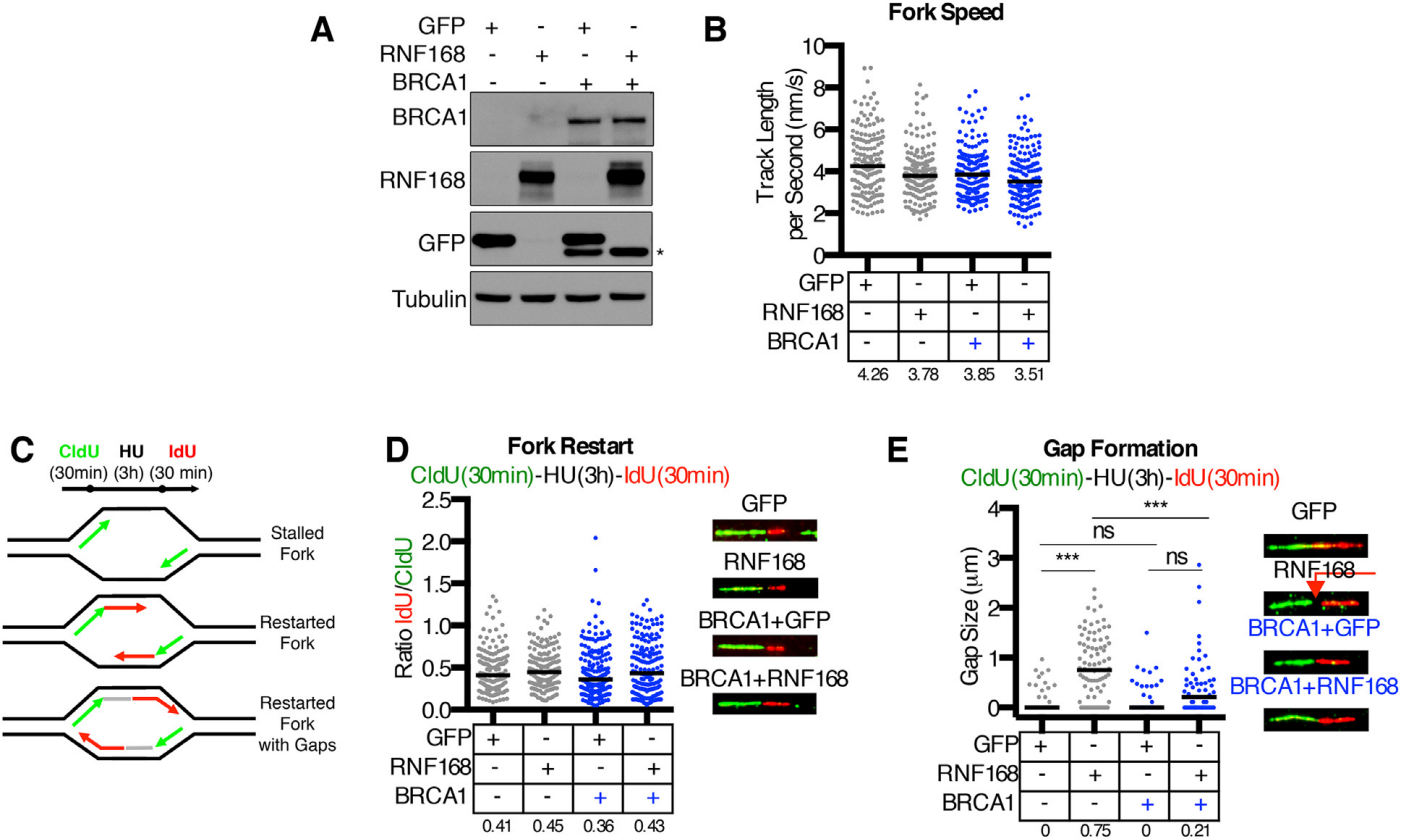
Effects of ectopic RNF168 overexpression at stalled replication forks. (**A**) Doxycycline (dox)-inducible GFP and RNF168 overexpression in SUM1315MO2 parental and BRCA1 add back assessed by Western blotting after 72 h of dox addition to culture media. Asterisk, IRES-GFP used to select BRCA1 positive cells. (**B**) GFP and RNF168 expressing SUM1315MO2 ± BRCA1 cells were assessed for replication fork speed by incubating exponentially growing cells with 50 μM CldU for 30 min. DNA fibers were stained using immunofluorescence with a CldU-specific antibody. A minimum of 150 CldU fiber tract lengths were measured per sample and speed determined using the 30 min CldU incubation time. Calculated speeds for individual replications forks are shown and black bar indicates median values (median numbers are also shown below). (**C**) Cartoon showing potential replication fork restart outcomes from cells preincubated with 50 μM CldU for 30 min, washed and treated with 4 mM HU for 3 h, followed by washing and incubation with 250 μM IdU for 30 min. (**D**) Replication fork restart in SUM1315MO2 ± BRCA1 cells expressing either GFP or RNF168 using treatment conditions described in (C). A minimum of 150 replication forks were measured and DNA fiber lengths are presented as the IdU/CldU length ratio, black bar indicates median values (median numbers are also shown below). Inset, representative fibers. (**E**) Restarted forks from (D) were defined as forks with an IdU tract length >5 μm and were further assessed for the presence of unlabeled gaps between CldU and IdU tracts. Individual gap and median gap size (black bar) are shown (numbers are also shown below). ***P < 0.001 or not significant (ns) *P* > 0.05 differences between samples. (Mann–Whitney test). Inset, representative fibers, red arrow indicates gap.

SUM1315MO2 cells harbor a hemizygous *BRCA1*^185delAG^ mutation and were utilized to overexpress ectopic RNF168 or a GFP control protein. Although SUM1315MO2 cells have the ability to express a BRCA1 RING domain-deficient (Rdd) protein under PARPi selection pressure (23), the parental cell line used in our current experiments had low/undetectable BRCA1-Rdd protein expression. SUM1315MO2 cells complemented with wild-type BRCA1 were used as a comparator (Figure 1A). We initially examined replication fork speed using CIdU labeled DNA tract length measurements. Minimal differences in fork speed were observed between GFP and RNF168 or overexpressing cells as well as BRCA1 deficient or add back cells (Figure 1B).

In replication fork restart assays, cells were pre-treated with CldU, followed by 3 h of hydroxyurea (HU) to induce fork stalling and subsequently released into IdU-containing media (Figure 1C). Using this approach, we did not detect differences in the efficiency of replication fork restart between ectopic GFP, RNF168 and BRCA1 protein expressing isogenic cell lines (Figure 1D). Intriguingly, we noticed an abundance of unlabeled gaps that separated the green and red fibers in RNF168 overexpressing BRCA1-deficient SUM1315MO2 cells, but were present at significantly lower numbers in BRCA1 add back cells (Figure 1E). Therefore, although RNF168 did not impact the rate of fork progression in unchallenged cells, unlabeled gaps arose during the HU incubation period, specifically in BRCA1-deficient cells that overexpress RNF168.

### RNF168-induced DNA synthesis occurs during HU treatment in the absence of BRCA1

**Figure 2.**
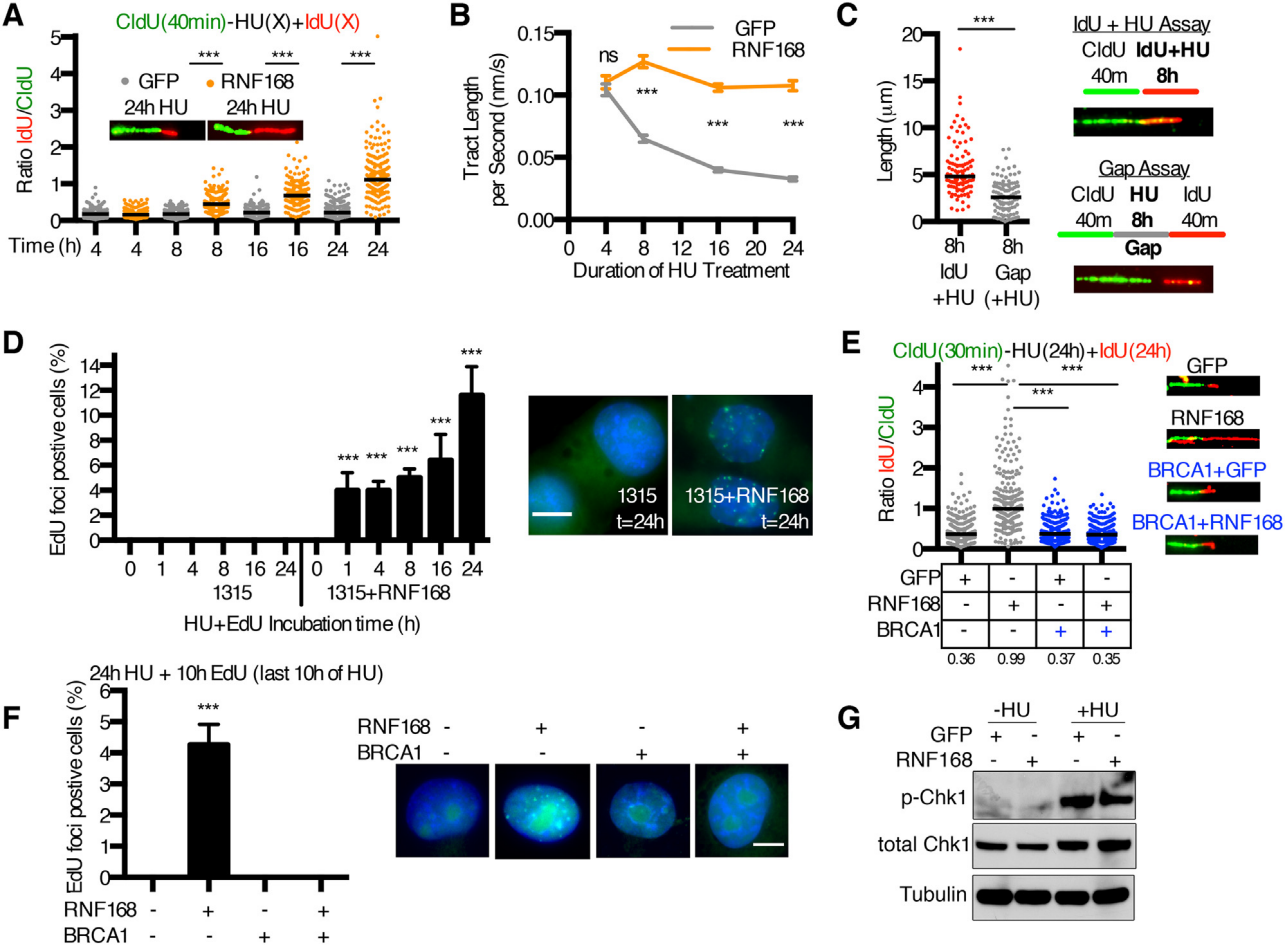
Ectopic RNF168 promotes DNA synthesis in the presence of HU. (**A**) GFP or RNF168 expressing SUM1315MO2 cells were incubated with CldU for 40 min following by increasing intervals of 4 mM HU and IdU. A minimum of 200 replication forks from two biological replicates were measured for each time point. Fiber lengths are presented as the IdU/CldU ratio, black bar indicates median values. ***P < 0.001 (Mann–Whitney test). Inset, representative fibers at the 24 h time point. See Supplementary Figure S1 for corresponding CldU and IdU tract length measurements. (**B**) Replication fork speed was assessed for data shown in (A). Mean ± S.E.M. fork speeds presented were generated by dividing the fiber length at each time point by the HU incubation time period. (**C**) DNA synthesis was assessed during an 8 h HU incubation in the presence and absence of IdU by measuring a minimum of 100 IdU labeled and unlabeled gap lengths, respectively. ***P < 0.001 (Mann–Whitney test). Inset, representative fibers. (**D**) SUM1315MO2 cells with and without RNF168 expression were co-treated with 10 μM EdU and 4 mM HU foci for the indicated times, and assessed for EdU foci formation. Nuclei were counterstained with DAPI and scored positive for EdU when five or more foci were detected. Mean ± S.D. foci positive cells are displayed (five fields of view/minimum of 200 cells/sample) and representative nuclei shown from the 24 h time point, 10 μm scale bar. ***P < 0.001 (two-tailed unpaired *t*-test comparing samples at the same time points). (**E**) GFP or RNF168 expressing SUM1315MO2 ± BRCA1 cells were incubated with CldU for 30 min followed by co-incubation with HU and IdU for 24 h then DNA fibers assessed. A combined minimum of 200 replication forks from two biological replicates were measured. Fiber lengths are presented as the IdU/CldU ratio, black bar indicates median values (numbers shown below). ***P < 0.001 (Mann–Whitney test). Inset, representative fibers. (**F**) SUM1315MO2 cells with ± BRCA1 and ± RNF168 expression were treated with 4mM HU for 24 h and 10 μM EdU was added during the last 10 h of the treatment period. Nuclei were scored positive when five or more EdU foci were detected. Mean ± S.D. foci positive cells are displayed (five fields of view/minimum of 200 cells/sample) and representative nuclei shown, 10 μm scale bar. ***P < 0.001 (two-tailed unpaired *t*-test comparing ± RNF168 samples). (**G**) GFP and RNF168 expressing SUM1315MO2 cells were treated with 4 mM HU for 8 h and subjected to Western blotting for phosphorylation at Ser317 of Chk1 (p-Chk1) and total Chk1.

Because color-labels were absent during HU treatment, we hypothesized that gaps may have been generated through ongoing DNA synthesis during the HU incubation period. To test this possibility, SUM1315MO2 parental cells that expressed either GFP control or RNF168 were incubated with CldU, followed by co-incubation with IdU and HU, and fiber lengths assessed at a range of time points. As expected, IdU-labeled fibers were absent or limited in length in GFP-expressing control cells over a 24-h HU incubation period. In contrast, IdU-labeled fibers that were adjoined to CldU fibers could be detected at 4 h, and gradually extended over the time course in RNF168 expressing cells (Figure 2A and Supplementary Figure S1). Notably, throughout the 24 h HU treatment period the rate of synthesis in SUM1315MO2+RNF168 cells remained consistent (Figure 2B), which was ∼30-fold slower than observed in unperturbed cells (Figure 1B). The presence of IdU moderately increased fork speed during HU treatment compared to measurements of unlabeled gaps (Figure 2C); possibly due to IdU partially compensating for the decreased nucleotide pool (24).

DNA synthesis was also measured by quantifying EdU-containing nuclear foci after cells were co-incubated with HU and EdU. Similar to DNA fiber assays, while no EdU foci could be detected in GFP cells, RNF168-expressing cells showed a gradual increase in the number of EdU foci-positive nuclei (Figure 2D). Strikingly, both DNA fiber lengthening and EdU foci were blocked in RNF168 overexpressing BRCA1 add-back cells (Figure 2E and F). Failure to activate ATR-CHK1 signaling has previously been shown to abrogate checkpoint-mediated fork stalling (25); however, we observed robust HU-induced phospho(Ser317)-CHK1 expression in GFP- and RNF168-expressing SUM1315MO2 cells (Figure 2G).

**Figure 3.**
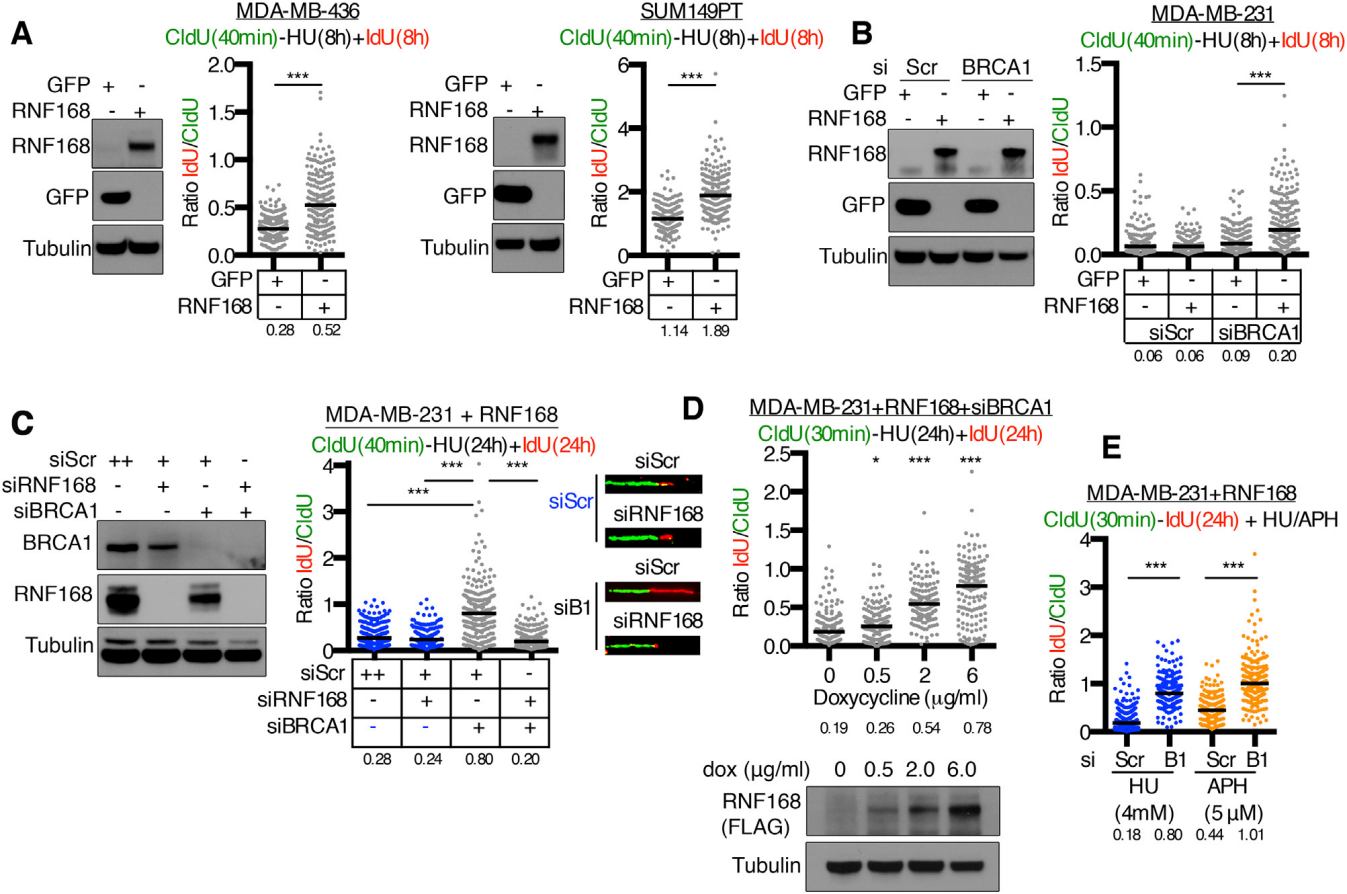
RNF168 overexpression supports synthesis in the absence of BRCA1. (**A**) *BRCA1* mutant MDA-MB-436 and SUM149PT cell lines expressing GFP or RNF168 were incubated with CldU for 40 min followed by an 8 h co-incubation of 4 mM HU and IdU. CldU and IdU containing DNA fiber lengths were assessed and presented as the IdU/CldU ratio, black bar indicates median values (numbers below). A minimum of 200 replication forks were measured. ***P < 0.001 (Mann–Whitney). (**B**) *BRCA1* wild-type MDA-MB-231 cells expressing GFP or RNF168 were treated with scrambled (Scr) or BRCA1 targeted siRNA then assessed as described in (A). (**C**) RNF168 expressing MDA-MB-231 cells were treated with scrambled (Scr), RNF168 or BRCA1 targeting siRNA then incubated with CldU for 40 min followed by co-incubation with HU and IdU for 24 h. Fiber lengths are presented as the IdU/CldU ratio, black bar indicates median values (numbers below). A combined minimum of 200 replication forks from two biological replicates were measured. ***P < 0.001 (Mann–Whitney). Inset, representative fibers. (**D**) MDA-MB-231 cells with doxycycline (dox)-inducible expression of RNF168 were cultured with the indicated concentrations of dox and treated with BRCA1 targeting siRNA. Cells were incubated with CldU for 30 min followed by co-incubation with HU and IdU for 24 h. Fiber lengths are presented as the IdU/CldU ratio, black bar indicates median values (numbers below). A minimum of 150 replication forks were measured. ***P < 0.001 (Mann–Whitney) compared to the no dox control. Inset, ectopic RNF168 expression was assessed by western blotting. (**E**) RNF168 expressing MDA-MB-231 cells were treated with BRCA1 targeting or scrambled (Scr) siRNA then incubated with CldU for 30 min followed by a 24 h co-incubation of IdU with either 4 mM HU or 5 μM aphidicolin (APH). Fiber lengths are presented as the IdU/CldU ratio, black bar indicates median values (numbers below). A minimum of 150 replication forks were measured. ***P < 0.001 (Mann–Whitney).

To determine if ectopic RNF168 overexpression promotes DNA synthesis in the presence of HU in additional cell lines, *BRCA1* mutant MDA-MB-436 and SUM149PT cells were engineered to overexpress either GFP or RNF168. Here, RNF168 overexpressing cells demonstrated increased DNA fiber lengths in the presence of HU compared to GFP expressing control cells (Figure 3A). Furthermore, *BRCA1* wild-type MDA-MB-231 cells that overexpress GFP or RNF168 were generated and subject to scrambled or BRCA1-targeting siRNA. DNA synthesis was observed in the presence of HU only in RNF168 overexpressing and BRCA1 siRNA treated cells (Figure 3B). Importantly, co-transfection with RNF168-targeting siRNA reversed BRCA1 siRNA-induced fiber lengthening (Figure 3C). Moreover, BRCA1 siRNA treated cells incubated with increasing concentrations of dox showed a dose-dependent increase in RNF168 expression that reflected the activation of DNA synthesis (Figure 3D). Similar to HU treatments, DNA synthesis events were also observed in response to fork stalling induced by aphidicolin treatment in RNF168 overexpressing and BRCA1 siRNA treated cells (Figure 3E). Of note, RNF168 expression did not affect cellular PARPi, HU or cisplatin sensitivity (Supplementary Figure S2). All in, ectopic RNF168 overexpression supported DNA synthesis in the presence of concentrations of HU or aphidicolin that would usually block fork progression in BRCA1 deficient cells.

### Ectopic RNF168 promotes BIR in the absence of HR

**Figure 4.**
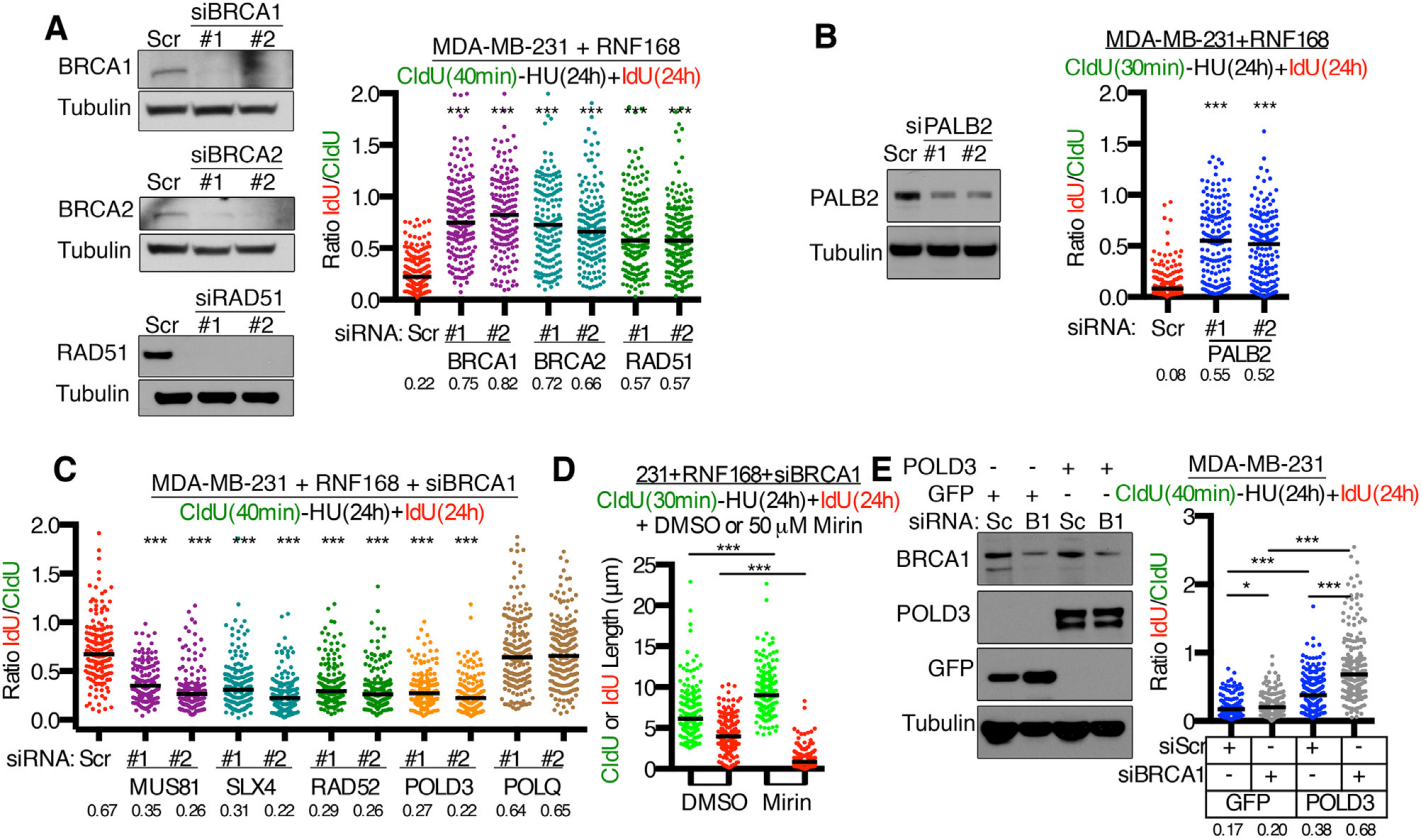
BIR proteins support fork progression. (**A**) RNF168 expressing MDA-MB-231 cells were treated with the indicated siRNAs then incubated with CldU for 40 min followed by co-incubation with HU and IdU for 24 h. Fiber lengths are presented as the IdU/CldU ratio, black bar indicates median values (numbers below). A combined minimum of 150 replication forks from two biological replicates were measured. ***P < 0.001 (Mann–Whitney) compared to the Scr siRNA treated control. Inset, Western blots showing the effects of two independent siRNAs on protein expression. (**B**) RNF168 expressing MDA-MB-231 cells treated with PALB2 targeting or scrambled (Scr) siRNA then incubated with CldU for 30 min followed by co-incubation with HU and IdU for 24 h. Fiber lengths are presented as the IdU/CldU ratio, black bar indicates median values (numbers below). A minimum of 150 replication forks were measured for each condition. ***P < 0.001 (Mann–Whitney test) compared to the Scr siRNA treated control. Inset, western blots showing the effects of two independent siRNAs on protein expression. (**C**) RNF168 expressing MDA-MB-231 cells were treated with the indicated siRNAs and assessed as described in (A). A minimum of 150 replication forks were measured for each condition. ***P < 0.001 (Mann–Whitney test) compared to the Scr siRNA treated control. (**D**) MDA-MB-231 cells expressing RNF168 were treated with *BRCA1* targeted siRNA, then incubated with CldU for 30 min followed by HU, IdU and 50 μM mirin or DMSO. Fiber lengths for CldU tracts are shown in green and IdU tract lengths shown in red, black bar indicates median values. A minimum of 150 tract lengths were measured for each condition. ***P < 0.001 (Mann–Whitney test) DMSO compared to mirin treated cells. (**E**) MDA-MB-231 cells were transduced to express GFP or POLD3, treated with scrambled (scr) or BRCA1-targeted siRNA, and subject to western blotting for the indicated proteins. DNA fibers were assessed as described in (A). A minimum of 150 replication forks were measured per condition. *P < 0.05, ***P < 0.001 (Mann–Whitney test).

To determine if the ability of ectopic RNF168 to promote DNA synthesis in the presence of HU was specific to BRCA1 depletion, we used RNAi targeting additional key HR proteins. Here, BRCA2 and RAD51 siRNA produced similar effects on DNA fiber length extension as BRCA1 siRNA in MDA-MB-231 cells (Figure 4A). Additionally, RNF168 has been shown to promote BRCA1-independent PALB2-BRCA2-RAD51 loading. However, we found that PALB2 siRNA also induced DNA synthesis (Figure 4B), suggesting that the RNF168–PALB2 complex did not contribute to this phenotype.

Because the concentration of HU used in these experiments (4 mM) is expected to induce DSBs at replication forks as opposed to fork slowing or stalling (26), we assessed the impact of depletion of the fork processing nucleases MUS81 and SLX4 (27). Here, co-depletion of BRCA1 and MUS81 or SLX4 abrogated the DNA fiber extension observed during HU with BRCA1 depletion alone in RNF168 overexpressing MDA-MB-231 cells (Figure 4C). Thus, RNF168-induced fiber lengthening likely results from the nucleolytic processing of stalled forks that subsequently restart, as opposed to the slowed progression of unperturbed forks. In support of this notion, inspection of individual fiber lengths showed that CldU tracts shortened with HU between 0 and 4 h, consistent with fork degradation. However, there were no further decreases in CldU lengths between 4 and 24 h HU (Supplementary Figure S3A). By 4 h of HU, some IdU tract lengths increased and lengthening was readily detectable at 24 h (Supplementary Figure S3B). These data suggest that, in BRCA1 deficient cells overexpressing RNF168, fork degradation initially occurs between 0 and 4 h of HU, but DNA synthesis restarts between 4 and 24 h and is reflected in IdU/CldU ratios (Supplementary Figure S3C). As expected, mirin treatment blocked CldU tract degradation, but also prevented IdU tract length elongation (Figure 4D), indicating that fork degradation was required for DNA synthesis restart.

BIR or LTGC are replication-dependent DSB repair events that involve extensive DNA synthesis (7,11), and can occur independently of BRCA1, BRCA2 and RAD51 (6,8). Therefore, we hypothesized that continued DNA synthesis may result from a BIR/LTGC-like event. RNAi-mediated depletion of RAD52 and POLD3, which are required for BIR (11), blocked RNF168 overexpression and BRCA1 siRNA-induced fork progression (Figure 4C). However, POLQ-targeting siRNA, a polymerase that is essential for MMEJ repair and possibly termination of BIR/LTGC (8), had no effect on BRCA1 depletion-induced fork progression (Figure 4C).

To confirm that BIR factors were responsible for DNA fiber lengthening, we overexpressed the Polδ subunit POLD3, which is required for BIR, in MDA-MB-231 cells (11,28). Here, POLD3 phenocopied RNF168 overexpression, with fork progression occurring in BRCA1 siRNA-treated cells (Figure 4E). These results indicate that in the absence of HR, RNF168 promotes DNA synthesis in the presence of HU in a manner that is dependent on the nucleases MUS81 and SLX4, as well as RAD52 and POLD3, potentially indicative of a BIR/LTGC-like DNA synthesis event.

### Ubiquitinated γH2AX stimulates DNA synthesis

**Figure 5.**
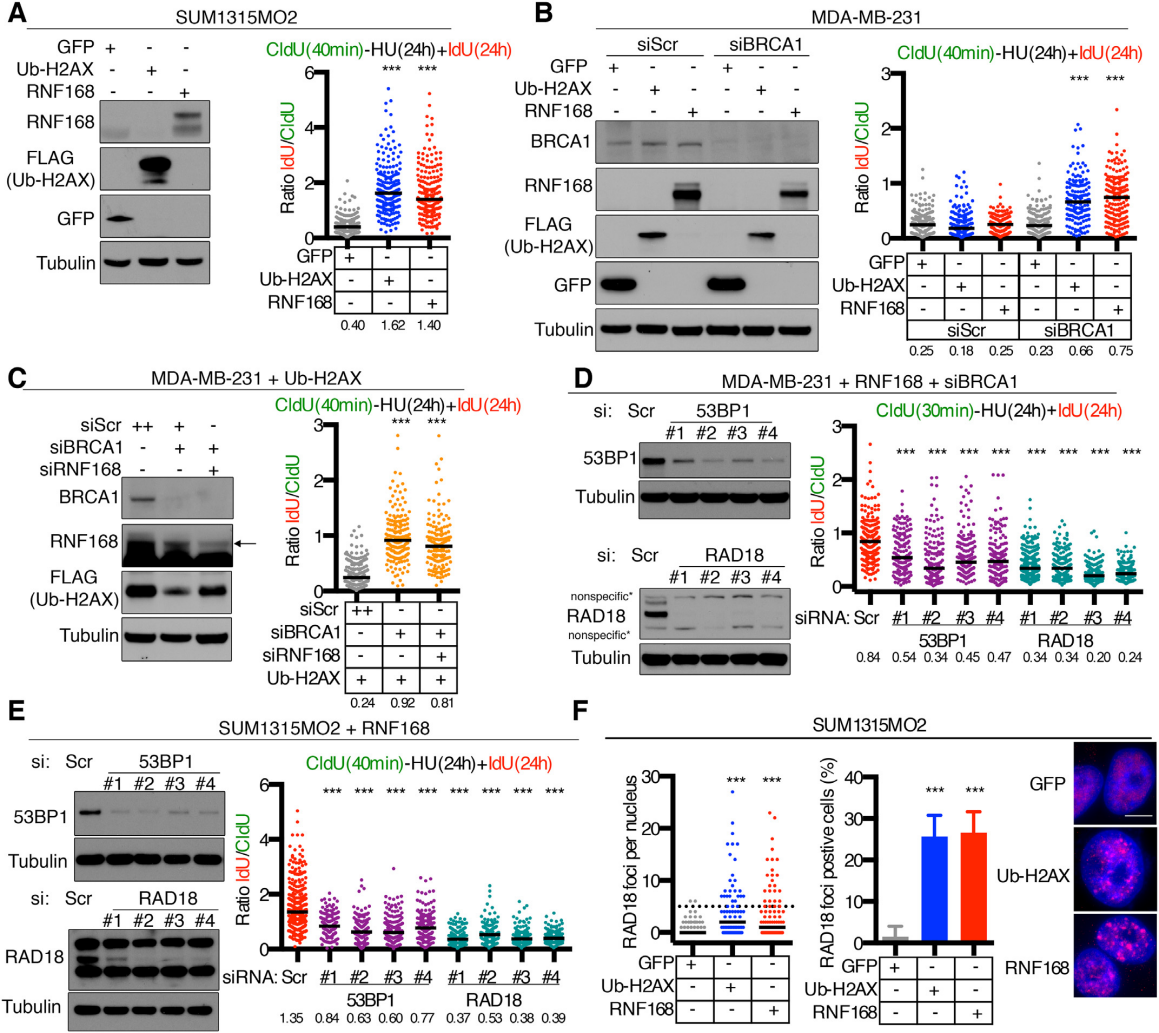
DNA fiber extension requires ub-γH2AX. (**A**) Dox-inducible GFP, ub-H2AX, RNF168 SUM1315MO2 cells were incubated for 40 min with CldU followed by a coincubation of HU and IdU for 24 h. IdU and CldU containing DNA fiber lengths were assessed and presented as the IdU/CldU ratio, black bar indicates median values (median numbers are also shown below). A combined minimum of 200 replication forks from two biological replicates were measured. ***P < 0.001 (Mann–Whitney test) compared to the GFP expressing control. (**B**) GFP, ub-H2AX and RNF168 expressing MDA-MB-231 cells treated with scrambled (Scr) or BRCA1-targeted siRNA were assayed as described in (A). A minimum of 150 replication forks were measured for each condition. ***P < 0.001 (Mann–Whitney test) compared to the scrambled siRNA treated control. (**C**) Dox-inducible ub-H2AX expressing MDA-MB-231 cells were treated as described in (A) as well as with the indicated siRNAs. A combined minimum of 150 replication forks from two biological replicates were measured. ***P < 0.001 (Mann–Whitney test) compared to the scrambled control. (**D**) Dox-inducible RNF168 expressing MDA-MB-231 were treated with the indicated siRNAs and subjected to western blotting. Fibers were examined as described in (A). A minimum of 150 replication forks were measured for each condition. ***P < 0.001 (Mann–Whitney test) compared to the scrambled control. Insets, representative fibers and Western blots. (**E**) Dox-inducible RNF168 expressing SUM1315MO2 cells were treated with siRNA targeting the indicated proteins and assessed for DNA synthesis as in (A). A minimum of 150 replication forks were measured for each condition. ***P < 0.001 (Mann-Whitney test) compared to the scrambled siRNA treated control. Insets, representative fibers and Western blots. (**F**) SUM1315MO2 cells expressing GFP, ub-H2AX, RNF168 were assessed for RAD18 foci formation after a 24 h 4 mM HU treatment. The median number of foci per nucleus are indicated with a black bar (left) and number of foci positive cells are presented as mean ± S.D. (right). A minimum of 100 nuclei were evaluated and nuclei were scored as positive if five or more foci were detected. ***P < 0.001 (two-tailed unpaired *t*-test).

RNF168 directly mono-ubiquitinates γH2AX at K13/15 within chromatin surrounding DNA breaks (13,14). To determine if RNF168-driven DNA synthesis resulted from γH2AX mono-ubiquitination, we expressed a ubiquitin N-terminal H2AX (ub-H2AX) fusion cDNA, previously shown to mimic K13/15 ub-γH2AX (29). Here, ub-H2AX induced DNA synthesis in the presence of HU in a manner identical to RNF168 overexpressing SUM1315MO2 (Figure 5A) and MDA-MB-231 cells (Figure 5B). Furthermore, ub-H2AX bypassed the requirement for RNF168 and promoted replication fork progression in MDA-MB-231 cells with BRCA1 and RNF168 co-depletion (Figure 5C). K13/15-ubiquitin-γH2AX is a binding module for 53BP1 and RAD18 (19,30); thus, we examined the impact of depletion of ubiquitin binding proteins on fork progression. RNAi-mediated depletion of 53BP1 reduced fiber lengths in the presence of HU, likely due to re-wiring of repair toward the HR pathway. Interestingly, RAD18 siRNA was overall more effective than 53BP1 depletion at blocking DNA fiber length progression in RNF168 overexpressing SUM1315MO2 cells (Figure 5D) and BRCA1 siRNA treated MDA-MB-231 cells (Figure 5E). Moreover, RAD18 foci dramatically increased when RNF168 or ub-H2AX were overexpressed in HU-treated cells (Figure 5F). The above observations indicate that RNF168 likely initiates BIR through the recruitment of RAD18 to ubiquitinated γH2AX.

### A RAD18-SLF1 axis drives BIR-like DNA synthesis

**Figure 6.**
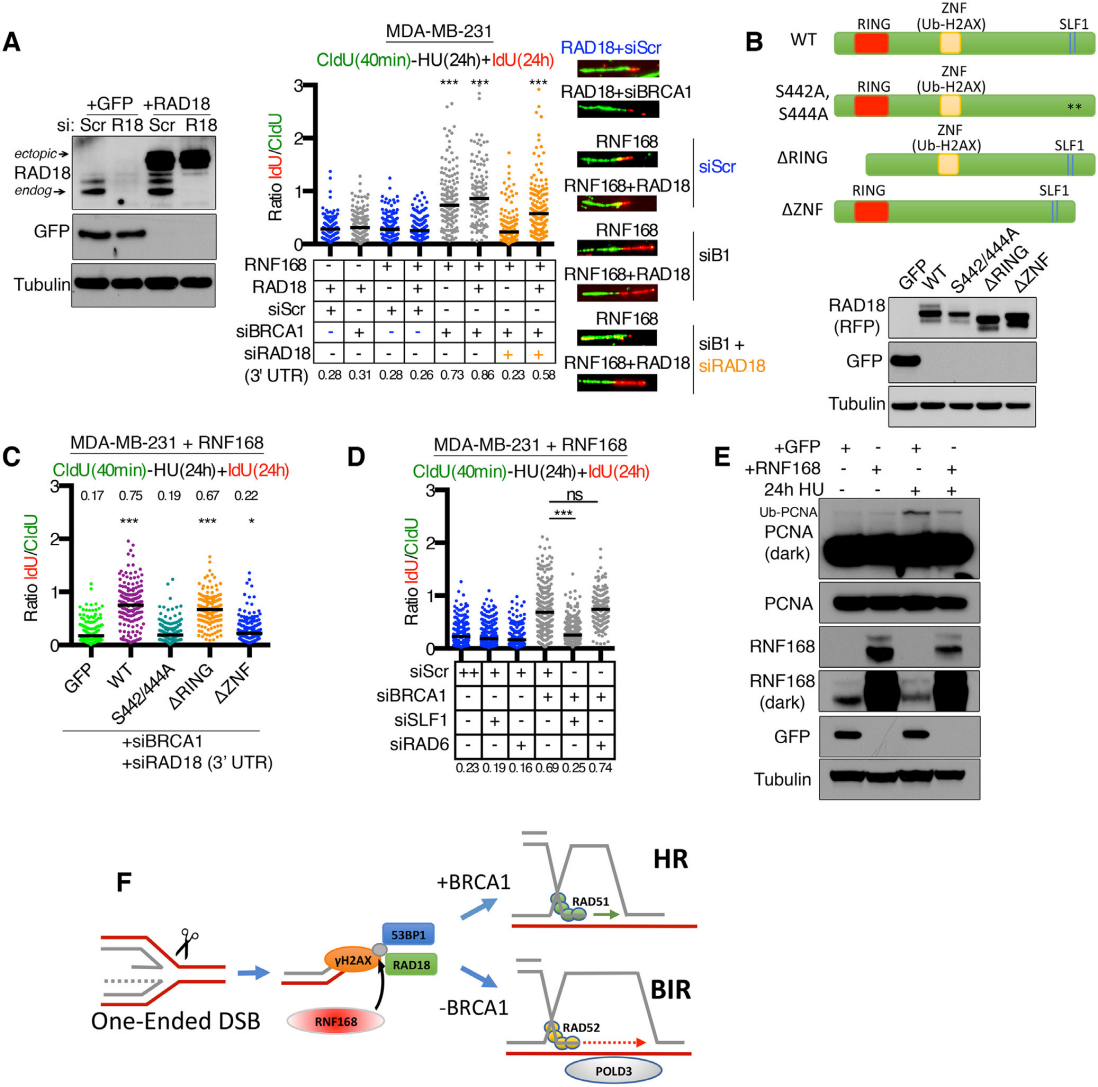
The RAD18–SLF1 interaction promotes DNA fiber progression. (**A**) MDA-MB-231 cells were transduced to express ectopic RAD18 or GFP and treated with scrambled siRNA or siRNA targeting the 3′ UTR of the endogenous RAD18. Lysates were subject to western blotting to confirm knockdown of endogenous RAD18 and reconstitution with ectopic RAD18 (*left*). MDA-MB-231 cells with ectopic RAD18 in combinations with dox-induced RNF168 and scrambled or BRCA1 targeted siRNA were incubated with CldU for 40 min followed by a 24 h co-incubation of HU and IdU. DNA fiber lengths were assessed and are presented as the IdU/CldU length ratio, black bar indicates median values (median numbers are also shown below). A minimum of 120 replication forks were measured for each condition. ***P < 0.001 (Mann–Whitney test) compared to the scrambled siRNA treated control. (**B**) The indicated RAD18 cDNA constructs and mutations were generated. RAD18 constructs and a GFP control were expressed in dox-inducible RNF168 MDA-MB-231 cells and assessed for expression by western blotting. (**C**) Cell lines from (B) were treated with siRNA targeting BRCA1 and RAD18 3′ UTR and fibers assessed as described in (A). A minimum of 150 replication forks were measured for each cell line. ***P < 0.001 (Mann–Whitney test) compared to the GFP expressing control. (**D**) Dox-inducible RNF168 expressing MDA-MB-231 cells were treated with the indicated siRNAs and fibers assessed as described in (A). A minimum of 200 replication forks were measured for each condition. ***P < 0.001 (Mann–Whitney test). (**E**) Dox-inducible GFP or RNF168 expressing MDA-MB-231 cells were treated with 4 mM HU or vehicle for 24 h and subjected to western blotting for the indicated proteins. (**F**) One-ended DSBs arising at HU-induced stalled replication forks activate RNF168, which subsequently localizes 53BP1 and RAD18 to ub-H2AX. In the presence of BRCA1, HR repair ensues. In the absence of BRCA1, 53BP1 inhibits DNA end resection while RAD18 promotes BIR.

To gain mechanistic insight into the role of RAD18, we expressed ectopic wild-type and mutant RAD18 proteins in MDA-MB-231 cells that also expressed ectopic RNF168. Cells were subsequently treated with BRCA1 siRNA as well as RAD18 3′UTR-targeting siRNA to deplete endogenous RAD18 but leave ectopic RAD18 intact (Figure 6A). The ZNF domain is required for ubiquitin binding, and RAD18^ΔZNF^ was previously shown to be unable to form IRIF (19). RAD18 heterodimerizes with RAD6 via respective RING domains, whereas RAD18 phosphorylated at S442/S444 binds to the SLF1 BRCT domain and forms a complex that is physically distinct from the RAD18-RAD6 ubiquitin ligase (31) (Figure 6B). The presence of RAD18^WT^ and RAD18^ΔRING^ restored DNA fiber lengths in the presence of HU and siRNA targeting endogenous RAD18, but RAD18^S442/444A^ and RAD18^ΔZNF^ proteins were both unable to rescue DNA fiber progression (Figure 6C). We further distinguished the impact of blocking either RAD18-SLF1 or RAD18-RAD6 activities by targeting SLF1 and RAD6 with siRNA and measuring fork progression. Here, in line with RAD18 mutant protein analyses, SLF1, but not RAD6 siRNA, blocked fork progression (Figure 6D). Importantly, the RAD18–RAD6 complex ubiquitinates PCNA, and we did not observe substantial differences in PCNA ubiquitination in the presence of RNF168 (Figure 6E). All in, these data suggest that, rather than RAD18–RAD6, the RAD18–SLF1 complex mediates BIR-like DNA synthesis at stalled forks.

## DISCUSSION

RNF168 expression and signaling is deregulated in tumors and cancer cell lines by mechanisms that include increased mRNA expression, downregulation of TRIP12 and UBR5 proteases, as well as reduced expression of enzymes that de-ubiquitinate ub-H2AX (21,32–34). Because 53BP1 is recruited to RNF168-associated ubiquitin marks, an increase in RNF168 expression further decreases DNA end resection and HR activity in *BRCA1* mutant cells (20). Moreover, RNF168 overexpression-induced inhibition of DNA end resection presumably prevents downstream PALB2-BRCA2-RAD51 loading due to a lack of ssDNA substrate (35,36). When two-ended DSBs are not resected, NHEJ is likely to become dominant and repair breaks. However, at one-ended DSBs, where NHEJ is not active, we propose that RNF168 induces BIR/LTGC-like fork restart and DNA synthesis (Figure 6F). LTGC has been shown to occur at Tus/Ter-induced stalled replication forks in BRCA1 deficient cells (7), and may be responsible for the TD signature commonly observed in *BRCA1* mutant cancers (8).

RAD18 is another ubiquitin binding protein that is recruited to RNF168 generated K13/15-ub-γH2AX. RAD18 was shown to directly interact with SLF1 and subsequently target the SLF1/2-SMC5/6 cohesion complex to sites of ub-γH2AX at DNA lesions in an RNF168-dependent manner (31). The latter complex is distinct and physically separate from the RAD18-RAD6 heterodimer, which ubiquitinates PCNA and activates TLS. Interestingly, RAD18 promotes replication fork protection in *BRCA1* mutant cancers through the PCNA-TLS pathway (37). We found that, downstream of fork protection, the RNF168-induced-BIR/LTGC-like DNA synthesis event was dependent on RAD18-SLF1, and RAD6 activity was not required, with RNF168 expression also having no effect on PCNA ubiquitination.

In the absence of BRCA1, overexpression of POLD3 had a similar effect as RNF168 on DNA synthesis. POLD3 was also recently shown to contribute to restart replication forks in BRCA2-deficient cells (27). In contrast to BRCA1 and HR-induced fork restart, the RNF168-RAD18-POLD3 driven restart was capable of synthesizing DNA in the presence of a reduced pool of nucleotides, albeit at significantly reduced speeds. Another study showed a similar DNA synthesis event in *BRCA1/2* WT cells that were treated with 5 mM HU and subject to FANCD2 depletion. FANCD2 depletion resulted in failure to activate the ATR checkpoint, consequently replication continued in the presence of HU (38). By contrast, ATR activation was intact in RNF168 overexpressing cells, and DNA synthesis was dependent on the fork restart factors MUS81/SLX4, as well as BIR proteins RAD52 and POLD3, indicating that FANCD2 depletion and RNF168 overexpression induce DNA synthesis by distinct mechanisms.

Although we were unable to detect DNA fiber lengthening with endogenous RNF168 expression, BIR/LTGC was previously shown to be active in *BRCA1* mutant cells under basal conditions using reporter assays (7). Our experiments were carried out in the presence of HU, and removal activated global DNA synthesis, hindering our ability to identify BIR-induced DNA fibers. Competition between HR and BIR was shown in yeast (39); we now propose that the BRCA1 and RNF168 mutational and expression status determine repair pathway outcomes at stalled replication forks in mammalian cells.

## Supplementary Material

gkaa154_Supplemental_File
