# Supplementary material for: Ectopic RNF168 expression promotes break-induced replication-like DNA synthesis at stalled replication forks
Source: Nucleic Acids Res. 2020 Mar 17;48(8):4298–308. doi: 10.1093/nar/gkaa154 (PMC7192614; doi:10.1093/nar/gkaa154)
Supplement: gkaa154_Supplemental_File [file gkaa154_supplemental_file.pdf]

Supplemental Figures

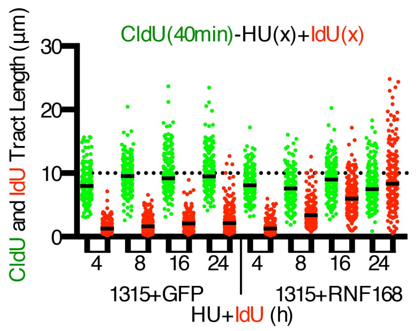

Figure S1. SUM1315MO2 tract lengths used to calculate ratios reported in Figure 2A.

GFP or RNF168 expressing SUM1315MO2 cells were incubated with CldU for 40 min following by increasing intervals of 4mM HU and IdU, as described in Figure 2A. Fiber lengths for CldU tracts are shown in green and IdU tracts in red, black bar indicates median values. These values were used to calculate the IdU/CldU ratios shown in Figure 2A.

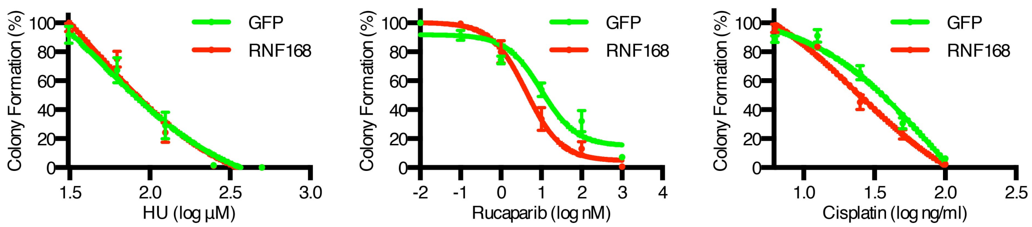

Figure S2. Effects of RNF168 expression on colony formation.

SUM1315MO2 cells expressing GFP or RNF168 were assessed for colony formation when seeded in the presence of the indicated concentrations of hydroxyurea (HU), the PARPi rucaparib, or cisplatin. Colony formation is expressed as a percentage of vehicle treated controls. Three biological replicates were performed for each assay.

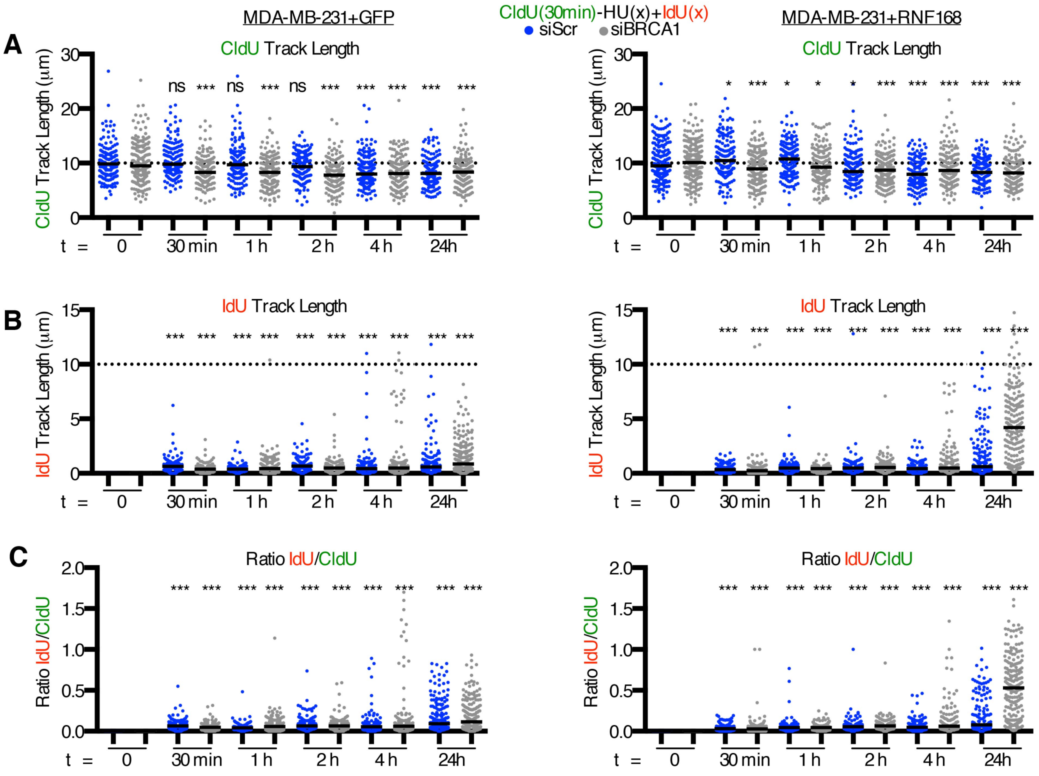

Figure S3. Tract lengths and ratios over 24h HU treatment.

(**A**) CldU tract lengths. GFP and RNF168 expressing MDA-MB-231 cells were treated with BRCA1 targeting or scrambled (Scr) siRNA then incubated with CldU for 30 min followed by a co-incubation of IdU and HU for the indicated times. CldU tract lengths are shown, black bar indicates median. A minimum of 150 replication forks were analyzed per condition. The dotted line marks 10 μm, the approximate median CldU tract length from a 30 min incubation with no subsequent HU incubation. ***$p < 0.001$, *$p < 0.05$, NS* $p > 0.05$ (Mann-Whitney test) comparing each sample to its corresponding t=0 time point.

(**B**) IdU tract lengths. Treatments are from (A), black bar indicates median length. The dotted line marks 10 μm for reference to the approximate value for 30 min of unimpeded synthesis. ***$p < 0.001$ (Mann-Whitney test) comparing each sample to its corresponding t=0 time point.

(**C**) The ratios of IdU/CldU tract lengths calculated from values reported in A and B are shown, black bar indicates median. ***$p < 0.001$ (Mann-Whitney test) comparing each sample to its corresponding t=0 time point.
